# Tetrel Bond between 6-OTX_3_-Fulvene and NH_3_: Substituents and Aromaticity

**DOI:** 10.3390/molecules24010010

**Published:** 2018-12-20

**Authors:** Ming-Chang Hou, Shu-Bin Yang, Qing-Zhong Li, Jian-Bo Cheng, Hai-Bei Li, Shu-Feng Liu

**Affiliations:** 1 The Laboratory of Theoretical and Computational Chemistry, School of Chemistry and Chemical Engineering, Yantai University, Yantai 264005, China; houmingchang2017@sina.com (M.-C.H.); emily_ipp@163.com (S.-B.Y.); cjb1962@vip.sina.com (J.-B.C.); 2 School of Ocean, Shandong University, Weihai 264209, China; 3 Shandong Key Laboratory of Biochemical Analysis, College of Chemistry and Molecular Engineering, Qingdao University of Science and Technology, Qingdao 266042, China; sliu@qust.edu.cn

**Keywords:** tetrel bonding, fulvene, substituents, aromaticity

## Abstract

Carbon bonding is a weak interaction, particularly when a neutral molecule acts as an electron donor. Thus, there is an interesting question of how to enhance carbon bonding. In this paper, we found that the –OCH_3_ group at the exocyclic carbon of fulvene can form a moderate carbon bond with NH_3_ with an interaction energy of about −10 kJ/mol. The –OSiH_3_ group engages in a stronger tetrel bond than does the –OGeH_3_ group, while a reverse result is found for both –OSiF_3_ and –OGeF_3_ groups. The abnormal order in the former is mainly due to the stronger orbital interaction in the –OSiH_3_ complex, which has a larger deformation energy. The cyano groups adjoined to the fulvene ring not only cause a change in the interaction type, from vdW interactions in the unsubstituted system of –OCF_3_ to carbon bonding, but also greatly strengthen tetrel bonding. The formation of tetrel bonding has an enhancing effect on the aromaticity of the fulvene ring.

## 1. Introduction

Fulvenes serve not only as synthetic precursors to naturally occurring compounds [1,2], but also as starting materials for the synthesis of novel substituted titanocenes, which are bio-organometallic anti-cancer drugs [3]. As an isomer of benzene, the structures and properties of fulvene are different from those of benzene. Fulvene is a non-alternant hydrocarbon, although it also displays a planar structure. Unlike benzene, this planar molecule is unstable both thermodynamically and kinetically, with very low resonance energy [4] and aromatic stabilization energy [5]. Despite fulvene being a non-aromatic molecule, the realization of its aromaticity has attracted much interest [6,7,8,9,10]. At its lowest excited states, fulvene is moderately aromatic, with a substantial contribution from the dipolar aromatic structure [6]. The electronic structure of the conjugated system in fulvene suffers substantial changes when exocyclic substitution occurs; its aromaticity increases by electron-donating substituents, and decreases by electron-withdrawing groups [7,8,9]. Interestingly, when a lithium atom approaches a face of the fulvene molecule, the fulvene moiety becomes aromatic, with an ‘aromatic’ NICS (nuclear independent chemical shift) value of −11 ppm [10]. When a fulvene ring is fused into the phenyl ring of an *O*-hydroxy Schiff base, the strength of an intramolecular hydrogen bond is tuned by the substituent at the exocyclic carbon in fulvene [11]. An electron-donating group at the exocyclic carbon in fulvene weakens the intramolecular hydrogen bond in some of the fulvene’s Schiff bases [12]. Cyano groups adjoined to the fulvene ring greatly increase the acidity of 6-OH-fulvene, resulting in organic superacids [13].

Recently, tetrel bonding, an attractive interaction between a Group IV atom and a Lewis base, has been attracting much attention [14,15,16,17,18,19,20]. Similarly to hydrogen and halogen bonds, tetrel bonding has broad applications in supermolecular materials [21,22,23] and chemical reactions [24,25,26]. Owing to the greater electronegativity and lower polarization, carbon atoms seldom engages in tetrel bonding, wherein it is also called a carbon bond if the carbon atom acts as a Lewis acid. Even so, carbon bonding has been observed in different systems [27,28,29,30,31,32,33,34,35,36,37,38,39,40,41,42,43]. A weak Ar···C interaction was firstly detected in an Ar···propargyl alcohol complex by microwave spectroscopy and ab initio calculations [27]. Mani and Arunan then performed a theoretical study of carbon bonding in complexes of methanol and methyl fluoride, where a methyl group is adjoined with an electron-withdrawing group, and found that in some neutral bases the acidity of the carbon atom increases, and the interaction energy amounts to ~8 kJ/mol [28]. The ubiquity of such carbon bond was further witnessed in the solid state by means of Cambridge Structural Database (CSD) and charge density analyses [29]. Similarly, the –CF_3_ group in para-substituted ArCF_3_ is able to participate in carbon bonding [30]. The –CF_3_ group in pyridine–CF_3_ and furan–CF_3_ is favorable for a weak hydrogen bond with NH_3_, but the protonation on the N and O atoms in the pyridine and furan rings results in a moderate carbon bond with NH_3_ [31]. A similar protonation enhancing effect was also reported for the –CH_3_ group [32]. In addition to sp^3^-hybridized carbon being involved in carbon bonding, the sp^2^-hybridized carbon in C=C and C=O bonds also acts as a Lewis acid [33,34,35,36,37,38,39,40,41]. Moreover, the sp^3^-hybridized carbon forms a weaker carbon bond than does the sp^2^-hybridized carbon in most cases. How can a carbon bond be strengthened when a sp^3^-hybridized carbon is acting as a Lewis acid?

It has been demonstrated that the hydroxyl proton of 6-OH-fulvene is acidic, and that its acidity increases greatly when cyano groups adjoin to the fulvene ring [13]. Inspired by these findings, in this study, the hydroxyl H atom of 6-OH-fulvene was changed to –CX_3_ (X = H and F), which was able to form a stronger carbon bond with NH_3_. In order to compare the strength of the tetrel bonds formed by different tetrel atoms, the –CX_3_ group in 6-OCX_3_-fulvene was also changed to –SiX_3_ and –GeX_3_. The four H atoms in the 2–5 positions of fulvene were then replaced by four cyano groups to enhance the acidity of CF_3_ and SiF_3_. For comparison, the corresponding complexes of PhOTX_3_ and HOTX_3_ (T = C, Si, and Ge) were also studied. We focused on the effect of both TX_3_ and the formation of tetrel bonds on the aromaticity of the fulvene ring.

## 2. Theoretical Methods

The second-order Moller-Plesset perturbation theory (MP2) method was utilized to optimize the structures of the complexes and monomers, using the aug-cc-pVTZ basis set. Frequency calculations were performed at the same level, to ensure they were at minima on the potential energy surfaces. This level of the theory has been often used to study tetrel-bonded complexes [15,16,25,31]. The stability of the complex was estimated, using the interaction and binding energies, by the supramolecular method (subtracting the energies of the monomers from the energy of the complex). Both interaction and binding energies were corrected for the basis set superposition error (BSSE) with the counterpoise method proposed by Boys and Bernardi [44]. For the interaction energy, the geometry of the monomer was the one in its complex, while for the binding energy, in contrast, the monomers were fully optimized. The magnitude of their difference is the deformation energy (DE), which was used to measure the deformation of both monomers as they came close to each other and formed the complexes. All calculations were carried out using the Gaussian 09 package of codes [45].

The molecular electrostatic potentials (MEPs) were calculated on the 0.001 electrons bohr^−3^ isosurface at the MP2/aug-cc-pVDZ level, using the WFA-SAS program [46]. The topological parameters of the interaction, including electron density, its Laplacian, and the energy density at the bond critical point (BCP), were analyzed by the AIM2000 [47] program. The natural bond orbital (NBO) analysis was carried out at the HF/aug-cc-pVTZ level, while the charge transfer and second order perturbation energy were obtained by NBO3.1 version [48] implemented in the Gaussian 09 program. The orbital interaction contribution was obtained by means of an analysis of natural orbital for chemical valence (NOCV), using the ADF program [49]. Interaction energy was decomposed by the LMOEDA method [50] at the MP2/aug-cc-pVTZ level, using the GAMESS program [51].

The NICS(1)_zz_ index [52] was calculated at the MP2/aug-cc-pVDZ level as the value of ZZ component of magnetic shielding tensor (taken with negative sign) at the point located 1 Å above a ghost atom located in the geometric center of the fulvene ring.

## 3. Results and Discussion

### 3.1. MEPs of 6-OTX_3_-Fulvene

**Figure 1:**
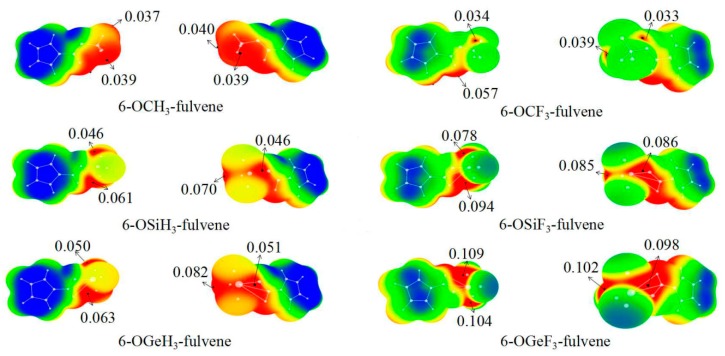
MEP maps of 6-OTX_3_-fulvene on the 0.001 electrons bohr^−3^ isosurface. Color ranges, in au, are: red, >0.03; yellow, 0.03–0.02; green, 0.02–0.00; blue, <0.

The MEP maps of 6-OTX_3_-fulvene with two views are presented in Figure 1. It is evident from Figure 1 that four σ-holes were found on the surface of the –TX_3_ (T = C, Si, and Ge) group. The MEP values of the four σ-holes varied between the different –TX_3_ groups. For the –TH_3_ (T = C, Si and Ge) group, the σ-hole along the O–T bond had the largest MEP, followed by the one along the H–T bond, which was coplanar with the C(sp^2^)-H bond. The MEP values of the left two σ-holes were approximately equal, being attributed to the C_s_ symmetrical geometries of 6-OTH_3_-fulvene. The F substitution in 6-OTF_3_-fulvene caused a complicated change in the order of the σ-hole magnitude, which was primarily due to the conformation change of the –TF_3_ group. The MEP of the σ-hole at the O–T end was not largest for the –TF_3_ (T = Si and Ge) group, and the location of the largest σ-hole was different for the –SiF_3_ and –GeF_3_ groups. The σ-holes of the –CX_3_ groups had a similar distribution to that of the –TH_3_ (T = Si and Ge) groups. It was noted that the values of 0.037 a.u. and 0.039 a.u. of –CH_3_ group corresponded to the three H atoms of this group, while the value of 0.057 au of –CF_3_ group corresponded to the H atom of C(sp^2^)–H bond.

The MEP of the σ-hole at the O–T end is listed in the last column of Table 1. It is evident that the MEP of this σ-hole showed an increasing trend from C to Si to Ge with the increase of the T atomic mass, due to the lower electronegativity and greater polarizability of the heavier T atom. Furthermore, with the F substituent, the σ-hole was enlarged, owing to the electron-withdrawing ability of F atoms, with the exception of 6-OCF_3_-fulvene, where it was slightly smaller than that of 6-OCH_3_-fulvene.

### 3.2. Geometries and Interaction Energy of Complexes

**Figure 2:**
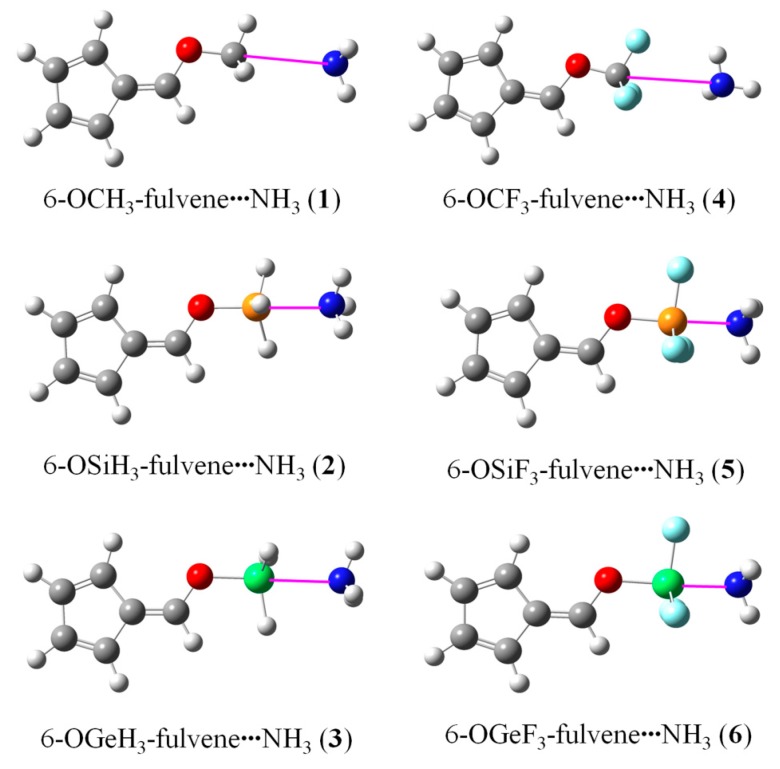
Optimized structures of the 6-OTX_3_-fulvene···NH_3_ complexes.

Figure 2 shows the optimized structures of the 6-OTX_3_-fulvene···NH_3_ complexes (T = C, Si, and Ge; X = H and F). In most complexes except 6-OCF_3_-fulvene···NH_3_, the angle ∠O–T···N was close to 180°, and three T–X bonds displayed a stagger conformation with three N–H bonds. In 6-OCF_3_-fulvene···NH_3_, two H atoms of NH_3_ pointed to the lone pair of two F atoms of 6-OCF_3_-fulvene. Even so, both molecules were combined through vdW interactions in 6-OCF_3_-fulvene···NH_3_. According to studies on the complexes of α/β-furanCF_3_ and p-PyCF_3_ with NH_3_ [31], they still had the capacity to form a tetrel bond interaction, as in 6-OCF_3_-fulvene···NH_3_. The Si···N and Ge···N distances were much shorter than the sum of their van der Waals radii (3.6 Å and 3.9 Å, respectively), and the F substituents further shortened their distance, especially for the Ge···N interaction.

The second column of Table 1 is the interaction energy (E_int_) of the complex calculated by the formula: E_int_ = E(complex) − E(monomer1) − E(monomer2), in which E(monomer) is the energy of the monomer within the complex structure. E_int_ was −9.93 kJ/mol in 6-OCH_3_-fulvene···NH_3_, which is twice as high as that of HOCH_3_···NH_3_. Thus, introducing a fulvene group to the carbon atom was an efficient method for enhancing the strength of tetrel bonding. In 6-OGeF_3_-fulvene···NH_3_, E_int_ was more negative than that in 6-OSiF_3_-fulvene···NH_3_. Without the F substituent, a reverse result was found, that is, the interaction in 6-OSiH_3_-fulvene···NH_3_ was stronger than that in 6-OGeH_3_-fulvene···NH_3_. This was inconsistent with the positive MEP of the Si and Ge atoms, indicating that electrostatic interaction is not the only dominant factor in the formation of a tetrel bond. Due to their strong electron-withdrawing ability, the F substituents in the tetrel donor molecule resulted in an increase of interaction energy by 87 and 112 kJ/mol for the Si and Ge complexes, respectively. It was found that the degree of interaction energy increased in that the effect of F substitution was more prominent for the Ge complex. The binding energy had a change similar to the interaction energy, although the former was smaller.

The geometrical deformation of tetrel donor molecules is often observed in tetrel-bonded complexes [53], where the tetrel atom is inclined to form a pentahedral structure, and thus to accommodate the approaching base. This geometrical deformation is measured by deformation energy (DE), which is the energy required to distort the interacting monomers from their equilibrium structures to the geometries in the complex. DE is negligible in 6-OCH_3_-fulvene···NH_3_ and 6-OCF_3_-fulvene···NH_3_, but is very large in the Si and Ge complexes, up to 99 kJ/mol in 6-OSiF_3_-fulvene···NH_3_. Dependent on the nature of the tetrel donor, the value of DE contributes to 24–72% of the interaction energy. It is evident from Table 1 that the silicon donor had larger DE than the Ge analogue. Furthermore, the F substituents in both Si and Ge tetrel donors led to a significant larger DE compared to that in 6-OTH_3_-fulvene···NH_3_.

The O–T bond was elongated during the formation of the complexes. The O–T bond elongation increased in the order of C < Ge < Si for a given –OTH_3_/–OTF_3_ group. This order was consistent with the interaction energy in 6-OTH_3_-fulvene···NH_3_, but an inconsistency was found in 6-OTF_3_-fulvene···NH_3_. This indicated that the O–T bond elongation is not a good indicator for the strength of a tetrel bond, different to that in H-bonding.

In order to get a deeper insight into the tetrel bond in 6-OTX_3_-fulvene···NH_3_, we introduced HOTX_3_ and PhOTX_3_ for comparison. We found that the binding modes with NH_3_ were the same for 6-OTX_3_-fulvene, HOTX_3_, and PhOTX_3_. The interaction strength was, in order, HOTX_3_ < PhOTX_3_ < 6-OTF_3_-fulvene, which was evidenced by the magnitudes of the intermolecular contact, the negative interaction energy, and the O–T bond elongation. This demonstrates that connecting with a benzene or fulvene ring is favorable to engage in a tetrel bond for a tetrel atom, especially with a fulvene group. It is apparent from Table 1 that the benzene or fulvene ring had a greater effect on the tetrel bond of an –OTH_3_ group than on that of an –OTF_3_ group. For example, when changing H to fulvene, the interaction energy of –OGeH_3_ complex increased by 175%, while that of –OGeF_3_ complex increased by 19%.

### 3.3. Substituents and AIM Analysis

**Figure 3:**
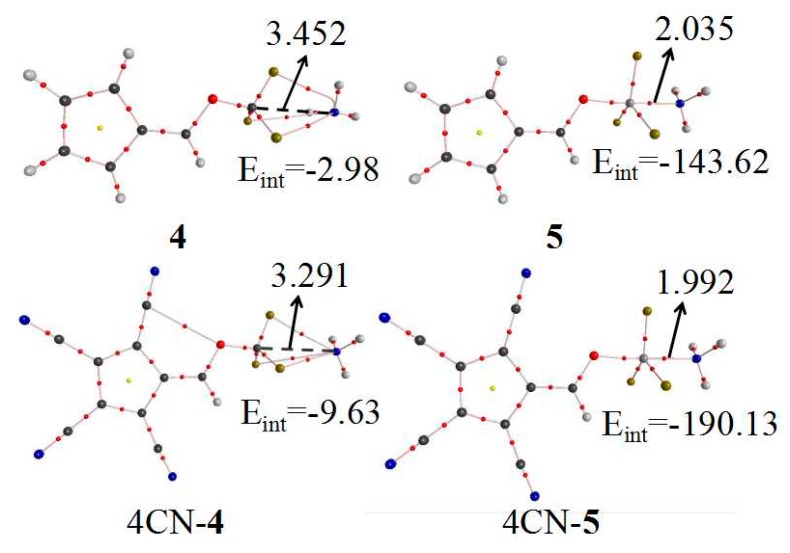
AIM diagrams before and after tetracyano substitution of 6-OTF_3_-fulvene···NH_3_ (T = C and Si). The units of distance and E_int_ are Å and kJ/mol, respectively.

The interaction mode between 6-OTX_3_-fulvene and NH_3_ was further determined by AIM analysis. Figure 3 shows the AIM diagrams of –CF_3_(4) and –SiF_3_(5). The other systems, except –CF_3_, were similar to 5, with only one BCP between the T atom and the N atom, confirming tetrel bond formation. Three BCP paths were found in 6-OCF_3_-fulvene···NH_3_, with one linear F···N path and two curved F···N paths in the vicinity of the N atom. The coexistence of three paths with a small interaction energy (~4 kJ/mol) implied that the interaction in this complex was a van der Waals force.

Table 2 presents the electron density, Laplacian, and total energy density at the BCP of the complexes, in which the mean values of the three F···N BCPs are given for 6-OCF_3_-fulvene···NH_3_. It confirms that the interaction type can be classified according to the sign of Laplacian and the total energy density [54]. Both were positive for 6-OCH_3_-fulvene···NH_3_ and 6-OCF_3_-fulvene···NH_3_, indicating, together with the small electron density, that both were weak close-shell interactions. For other complexes involving heavy tetrel donors, both the positive Laplacian and the negative total energy density demonstrated that the tetrel bond was a partially covalent interaction. The F substituents resulted in a further larger electron density, and thus gave rise to a stronger tetrel bond compared to the no substituent complexes. Moreover, the F substitution made the increase of electron density at the Ge···N BCP larger relative to the increase of electron density at the Si···N BCP. The enhancement of the electron density for the same T···N tetrel bond was accompanied by a greater Laplacian and more negative total energy density.

Through analysis of the optimized structure (Figure 2) and AIM maps, we can know that the –CF_3_ group of 6-OCF_3_-fulvene forms vdW interactions, not a tetrel bond, when it binds with NH_3_, like many molecules involving a –CF_3_ group [31]. Thus, a question is raised: is it possible for the –CF_3_ group of 6-OCF_3_-fulvene to form a tetrel bond with NH_3_? To answer it, the four H atoms on the five-membered ring of 6-OCF_3_-fulvene were replaced by the electron-withdrawing group, CN. The AIM analysis of the CN–substituent systems was calculated at the MP2/aug-cc-pVDZ level, together with the unsubstituted analogues—the corresponding AIM diagrams are also presented in Figure 3. Three intermolecular BCPs were found between the three F atoms of the –CF_3_ group and the N atom of NH_3_, which was also observed in the CF_4_···NCH tetrel-bonded complex [24]. This is clear evidence that the C···N tetrel bond exists in the complex of CN–substituent 6-OCF_3_-fulvene. Due to the strong electron-withdrawing effect of the CN group, the positive MEP on the σ-hole of –CF_3_ group was doubled from 0.039 a.u. to 0.072 a.u. Thus, the electron-withdrawing CN groups in the tetrel donor molecule can modulate the variation from vdW interactions to the tetrel bond. A similar effect was also realized by protonation of the tetrel donor molecule [31] or the methyl substituents of NH_3_, where the former similarly resulted in enlargement of the σ-hole of –CF_3_ group, and the latter not only led to an increase in basic electron donation, but also avoided the formation of the H-bond. The C···N separation was 3.291 Å in the CN–substituent complex, which was shortened by 0.161 Å relative to that in 6-OCF_3_-fulvene···NH_3_. As a consequence, the interaction energy increased from −2.98 to −9.63 kJ/mol, three times higher than that of vdW interactions.

To estimate the enhancing effect of CN substituents on the strength of tetrel bond, the four H atoms of the five-membered ring in 6-OSiF_3_-fulvene were also substituted by CN groups. This substitution caused an increase of the positive MEP on the σ-hole of the Si atom from 0.095 a.u. to 0.140 a.u., resulting in an increase in the acidity of Si atom. The tetrel bond was characterized by a Si···N BCP, with an electron density of 0.0679 a.u. and a negative total energy density. The Si···N distance was shortened from 2.035 Å to 1.992 Å, and the interaction energy increased from −143.62 to −190.13 kJ/mol. These data indicate that strong electron-withdrawing groups in tetrel donor molecules have a prominent enhancement effect on the strength of tetrel bonding.

### 3.4. NBO Analysis

Orbital interactions and charge transfer analysis were performed for the 6-OTX_3_-fulvene···NH_3_ complexes (Table 3). In 6-OCH_3_-fulvene···NH_3_, there was an orbital interaction of Lp_(N)_→σ*_C-O_, where Lp_(N)_ denotes the lone pair orbital of the N atom and σ*_C-O_ is the anti-bonding orbital of the C–O bond. This orbital interaction was very weak, with a perturbation energy of less than 6 kJ/mol. Additionally, the charge transfer was very small, consistent with the small interaction energy of this complex. Similarly, the orbital interaction Lp_(F)_→σ*_N-H_ in 6-OCF_3_-fulvene···NH_3_ was negligible (Table 3). This confirms its weak vdW interactions. For 6-OXF_3_-fulvene···NH_3_ (X = Si and Ge), there were two types of orbital interactions (Lp_(N)_→σ*_X-O_ and Lp_(N)_→σ*_X-F_), and the perturbation energy of Lp_(N)_→σ*_X-F_ listed in Table 3 is the sum of the three orbital interactions. The contribution of the Lp_(N)_→σ*_X-F_ orbital interaction was much larger than that of Lp_(N)_→σ*_X-O_, and both types of orbital interactions were stronger in 6-OGeF_3_-fulvene···NH_3_ than those in 6-OSiF_3_-fulvene···NH_3_, consistent with the interaction energy. Strong orbital interactions were related to high charge transfer (>0.16 e) in 6-OXF_3_-fulvene···NH_3_(X = Si and Ge). For 6-OXH_3_-fulvene···NH_3_ (X = Si and Ge), the Lp_(N)_→σ*_X-O_ orbital interaction was changed into Lp_(N)_→p*_X_ (the empty lone pair orbital on X atom), and Lp_(N)_→σ*_X-H_ was not changed. The Lp_(N)_→p*_X_ orbital interaction made a larger contribution than Lp_(N)_→σ*_X-H_, and both orbital interactions were stronger in 6-OSiH_3_-fulvene···NH_3_. Obviously, the F substituents significantly affected the type of orbital interaction as well as its strength.

**Figure 4:**
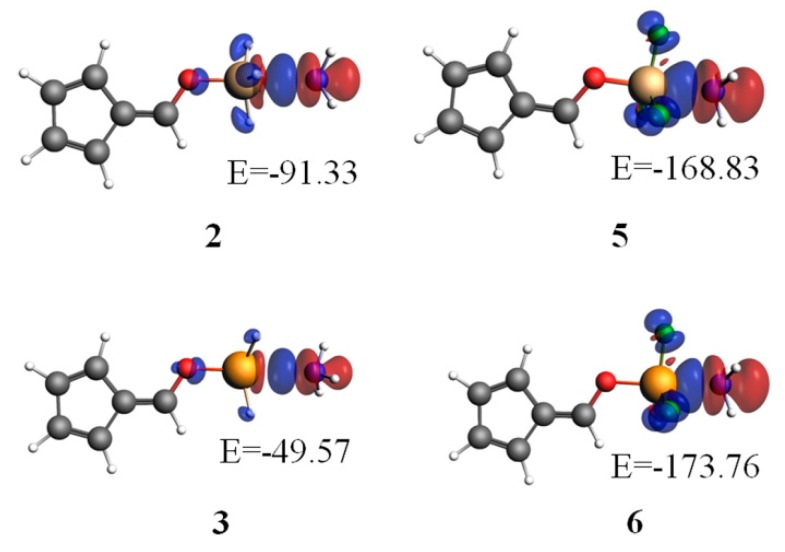
Plots of deformation densities of the pair-wise orbital interactions (Δρ) in 6-OTX_3_-fulvene···NH_3_ (T = Si, Ge; X = H, F) at the PBED3/TZ2P//MP2/aug-cc-pVTZ level. The associated orbital interaction energies are given in kJ/mol. The color code of the charge flow is red→blue, and the isovalue for Δρ(r) is 0.002 a.u.

In order to visually understand the contribution of orbital interactions to bonding energy, we performed an energy decomposition calculation, in conjunction with an analysis of natural orbital for chemical valence (NOCV), with the ADF 2008.01 program [49]. Figure 4 plots the deformation densities due to the pair-wise orbital interactions. The blue and red regions represent the increase and decrease of densities, respectively. It is obvious that NH_3_ was surrounded by red areas, while the group OTX_3_ was surrounded by blue regions. Thus, the charge flow shifted from NH_3_ (a base) to OTX_3_ (an acid). The largest contribution was from the orbital interaction between Lp_(N)_ and σ*_X-O_ in 6-OXH_3_-fulvene···NH_3_ (X = Si and Ge), while it was from that between Lp_(N)_ and σ*_X-F_ in 6-OXF_3_-fulvene···NH_3_ (X = Si and Ge). The former was larger for the SiH_3_ system, while the latter was larger for the GeF_3_ system. The stronger orbital interaction resulted in larger deformation densities. In TH_3_ complexes, the orbital interactions were relatively important; this can explain the stronger binding for Si than for Ge, although the positive MEP of the σ-hole for Ge was more positive. In TF_3_ complexes, the fluorines produced a stronger σ-hole, making the electrostatic interactions more important, and so the Ge was the stronger binder. This interplay between the electrostatic and orbital interactions has been previously suggested to be able to reverse the binding order [55].

### 3.5. Energy Decomposition Analysis

**Figure 5:**
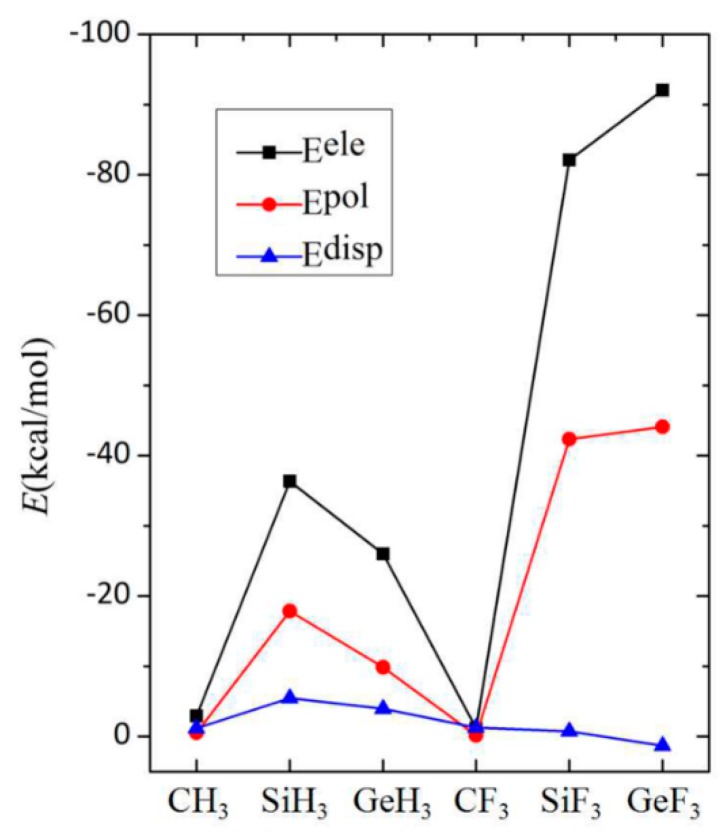
Dependence of three attractive energies on the TX_3_ group (T = C, Si, and Ge; X = H and F).

To gain a deeper insight into the characteristics of the tetrel bond in the complexes 6-OTX_3_-fulvene···NH_3_, its interaction energy was divided into five components: electrostatic energy (E^ele^), repulsion energy (E^rep^), exchange energy (E^ex^), polarization energy (E^pol^), and dispersion energy (E^disp^). Because both E^rep^ and E^ex^ cancel each other out, only three attractive terms (E^ele^, E^pol^, and E^disp^) are plotted in Figure 5, for ease of comparison. Each energy contribution was very small in the CX_3_ system, consistent with the weak characteristic of the interaction. In the SiX_3_ and GeX_3_ systems, the electrostatic energy was largest, followed by the polarization energy, and the dispersion energy was smallest. Both electrostatic and polarization contributions decreased from SiH_3_ to GeH_3_ systems, but increased from SiF_3_ to GeF_3_ systems (Figure 5), which illustrates the large effect of the F substituents on the nature of the tetrel bond. Owing to its smaller electronegativity, the Si atom in the SiH_3_ group was more easily polarized by a base than was the Ge atom in the GeH_3_ group. The greater polarization consequently resulted in a larger electrostatic interaction between both monomers, which was inconsistent with the magnitude of the positive MEP on the σ-hole along the O–T axis. The F substituents enlarged the MEP value of the σ-hole along the O–T axis. Thus, the electrostatic energy increased in the TF_3_ system, up to −90 kJ/mol in 6-OGeF_3_-fulvene···NH_3_, and was accompanied by a greater polarization. The large polarization energy means that the shape of the molecular orbital underwent a large change during the formation of the complexes, which is consistent with the deformation density of the pair-wise orbital interaction. This is consistent with the partially covalent nature of the tetrel bonding interactions of Si and Ge that was ascertained by analyzing the topological properties at the intermolecular BCPs.

### 3.6. Aromaticity of the Fulvene Ring

It has been confirmed that an electron-donating group at the exocyclic carbon atom of fulvene can increase its π-electron density [7,8,9]. Therefore, it is interesting to study the influence of a tetrel bond formation on the aromaticity of fulvene ring. Aromaticity is judged with NICS(1)_zz_ at the point located 1 Å above the fulvene ring center. A more negative NICS(1)_zz_ indicates a larger aromaticity. This value is collected in Table 4 for 6-OTX_3_-fulvene···NH_3_ and 6-OTX_3_-fulvene. It is evident that the NICS(1)_zz_ value was more negative in the complex than that in the isolated molecule, indicating that the formation of a tetrel bond resulted in an increase of aromaticity for the fulvene ring. It was confirmed that the charge transfer in the formation of the tetrel bond is from the lone pair on the N atom of NH_3_ to 6-OTX_3_-fulvene. This leads to an increase in the aromaticity and electron density of the fulvene ring. That is, the formation of a tetrel bond increased the aromaticity of the fulvene ring, similar with the electron-donating group at the exocyclic carbon atom. Furthermore, the increase of the NICS(1)_zz_ value was consistent with the charge transfer in SiX_3_ and GeX_3_ systems. For example, 6-OSiH_3_-fulvene···NH_3_ had a larger increase of NICS(1)_zz_ value and charge transfer than 6-OGeH_3_-fulvene···NH_3_.

## 4. Conclusions

The complexes of 6-OTX_3_-fulvene···NH_3_ (T = C, Si, Ge; X = H, F) were studied using theoretical calculations, with regard to geometrics, energetics, charge transfer, orbital interactions, and AIM parameters. The main conclusions are summarized as:

(1) The interaction energy of 6-OCH_3_-fulvene···NH_3_ was up to −10 kJ/mol, which was twice as high as that in HOCH_3_···NH_3_, thus, carbon bonding is enhanced when –OCH_3_ combines with fulvene ring.

(2) The σ-hole at the T–O bond end was larger in the –OGeX_3_ group than that in the –OSiX_3_ group, thus, –OGeF_3_ forms a stronger tetrel bond than –OSiF_3_. However, –SiH_3_ engaged in a stronger tetrel bond than –OGeH_3_, which is inconsistent with the magnitude of the σ-hole on both atoms. This inconsistency can be partly attributed to the stronger orbital interaction in 6-OSiH_3_-fulvene···NH_3_, accompanied by prominent distortion of –SiH_3_ group.

(3) The cyano groups adjoined to the fulvene ring increased the positive MEP on the σ-hole of –CF_3_ and –SiF_3_ groups, thus, the vdW interactions in 6-OCF_3_-fulvene···NH_3_ (4) were changed to carbon bonding in CN-4, and the interaction energy of tetrel bond increased by 32%, up to −190 kJ/mol in CN-5. Such CN substitution has a prominent effect on the type and strength of interactions.

(4) The formation of a tetrel bond was accompanied by a charge transfer from the lone pair on the N atom of NH_3_ to 6-OTX_3_-fulvene, thus, it resulted in an increase in the electron density on the fulvene ring and its aromaticity.

## Figures and Tables

**Table 1:** Interaction energy (E_int_, kJ/mol), binding energy (E_b_, kJ/mol), deformation energy (DE, kJ/mol), intermolecular distance (R, Å), ∠O–T···N (θ, deg), change of O–T bond length (∆*r*, Å), and the MEP maximum (V_max_, a.u.) of the σ-hole at the O–T bond end.

	E_int_	E_b_	DE	R	θ	∆*r*	*V*_max_
6-OTX_3_-fulvene···NH_3_							
–CH_3_	−9.93	−9.90	0.02	3.228	178.3	0.006	0.040
–SiH_3_	−50.13	−30.07	20.39	2.353	178.0	0.051	0.074
–GeH_3_	−39.30	−29.73	9.56	2.565	180.0	0.044	0.084
–CF_3_	−4.12	−3.97	0.15	3.498	169.3	0.003	0.039
–SiF_3_	−137.07	−38.04	99.03	2.052	176.9	0.063	0.095
–GeF_3_	−151.31	−70.36	80.94	2.074	176.7	0.046	0.113
HOTX_3_···NH_3_							
–CH_3_	−4.76	−4.70	0.06	3.384	174.9	0.003	0.019
–SiH_3_	−18.24	−14.89	3.35	2.792	175.9	0.017	0.051
–GeH_3_	−18.13	−15.89	2.24	2.872	175.5	0.018	0.059
–CF_3_	−3.55	−3.42	0.12	3.620	166.6	0.000	0.029
–SiF_3_	−101.03	−14.96	86.07	2.120	176.2	0.041	0.078
–GeF_3_	−126.75	−49.49	77.27	2.102	176.0	0.032	0.097
PhOTX_3_···NH_3_							
–CH_3_	−7.55	−7.43	0.12	3.302	176.0	0.004	0.028
–SiH_3_	−30.78	−20.56	10.22	2.539	177.6	0.032	0.057
–GeH_3_	−27.44	−21.96	5.48	2.697	178.1	0.030	0.067
–CF_3_	−3.66	−3.57	0.09	3.634	166.6	0.000	0.024
–SiF_3_	−115.29	−22.83	92.46	2.088	176.9	0.050	0.080
–GeF_3_	−134.93	−−56.92	79.17	2.094	175.8	0.038	0.096

**Table 2:** Electron density (ρ, a.u.), Laplacian (∇^2^ρ, a.u.), and total energy density (H, a.u.) at the intermolecular BCP.

	ρ	∇^2^ρ	*H*
**1**	0.006	0.026	0.002
**2**	0.033	0.079	−0.008
**3**	0.027	0.082	−0.008
**4**	0.004	0.019	0.001
**5**	0.063	0.251	−0.020
**6**	0.083	0.230	−0.033

**Table 3:** Charge transfer (CT, e) and second-order perturbation energies (*E*^(2)^, kJ/mol) in the complexes.

	CT	Orbitals	*E* ^(2)^	Orbitals	*E* ^(2)^
**1**	0.003	Lp_(N)_→σ*_C-O_	5.23		
**2**	0.093	Lp_(N)_→p*_Si_	186.72	Lp_(N)_→σ*_Si-H_	60.69
**3**	0.062	Lp_(N)_→p*_Ge_	129.91	Lp_(N)_→σ*_Ge-H_	34.44
**4**	−0.002	Lp_(F)_→σ*_N-H_	0.67		
**5**	0.163	Lp_(N)_→σ*_Si-O_	102.66	Lp_(N)_→σ*_Si-F_	296.53
**6**	0.186	Lp_(N)_→σ*_Ge-O_	134.76	Lp_(N)_→σ*_Ge-F_	401.61

Note: CT is the sum of the charge on all atoms of NH_3_.

**Table 4:** NICS(1)_zz_ in 6-OTX_3_-fulvene···NH_3_ and 6-OTX_3_-fulvene (in parentheses), as well as their difference (Δ); all are in ppm.

	NICS(1)_zz_	Δ
**1**	−6.0781 (−5.8122)	−0.2659
**2**	−6.7149 (−5.7074)	−1.0125
**3**	−6.9462 (−6.0684)	−0.8778
**4**	−5.2846 (−4.7814)	−0.5032
**5**	−6.0377 (−5.7074)	−0.3303
**6**	−6.2645 (−4.9984)	−1.2661
